# Expanding the Clinical Spectrum of Osteogenesis Imperfecta Type V: 13 Additional Patients and Review

**DOI:** 10.3389/fendo.2019.00375

**Published:** 2019-06-12

**Authors:** Yang-Jia Cao, Zhe Wei, Hao Zhang, Zhen-Lin Zhang

**Affiliations:** Metabolic Bone Disease and Genetic Research Unit, Department of Osteoporosis and Bone Diseases, Shanghai Jiao Tong University Affiliated Sixth People's Hospital, Shanghai, China

**Keywords:** osteogenesis imperfecta type V, IFITM5, mutation, phenotype, variability

## Abstract

Osteogenesis imperfecta (OI) is an inherited connective tissue disorder characterized by bone fragility and is characterized by clinical and genetic heterogeneity. Previous studies showed that the same mutation (c.−14C> T) of the *IFITM5* gene is responsible for autosomal dominant OI type V. However, the mutation has a variable expressivity. Clinical heterogeneity has been recognized in OI type V. In this study, we investigated 13 individuals with molecularly confirmed OI type V from seven Chinese families and explored the genotype-phenotype relationship. Increased callus formation is not observed in all individuals, and several novel clinical features were described: joint contractures (three individuals) and unexplained hip arthritis (six individuals). Significant clinical variability was observed even within families. Specific facial features were observed in six individuals from two families consistent with the facial features associated with OI type V reported so far in the literature. Interestingly, we report the process of hypertrophic callus formation in detail for the first time, and in five individuals with hyperplastic callus, increased erythrocyte sedimentation rate (ESR) and levels of C-reactive protein (C-RP) were measured, suggestive of inflammatory activation.

## Introduction

Osteogenesis imperfecta (OI) comprises a heterogeneous group of diseases characterized by susceptibility to bone fractures with variable severity and in most cases presumed or proven defects in collagen type I biosynthesis. The main characteristic is a liability to fractures. Secondary features are blue sclerae, dentinogenesis imperfecta, joint hypermobility, and short stature. Since the development of technology for gene detection, 24 genes have been identified to be related to OI with the majority of genetic causes influencing collagen type I biosynthesis.

In 2000, Glorieux et al. (1) reported several patients with distinctive clinical manifestations (hyperplastic callus formation [HC], radial head dislocation [RHD], radioulnar interosseous membrane ossification [RUIMO], and limitation in forearm rotation) and characteristic histopathology (irregular arranged lamellae and “mesh-like” appearance under polarized light) as having OI type V. Then, several teams confirmed that OI type V is consistently associated with a unique point mutation (c.-14C> T) in the 5′ untranslated region (UTR) of the *IFITM5* (interferon induced transmembrane protein 5) gene (2, 3). *IFITM5* is an osteoblast-specific gene associated with matrix mineralization that plays a putative role in bone formation and osteoblast maturation (4). As it turns out, the mutation (c.-14C> T) results in five amino acids (Met-Ala-Leu-Glu-Pro) being added to the N-terminus of the coding protein, BRIL, and alters its function (2). Until now, more than 100 individuals with OI type V have been described worldwide who carry the same mutation. The other reported mutation of *IFITM5* (c.119C> T) was identified as the cause of a severe variant of type VI OI (5). Although clinical and radiological abnormalities in OI type V have been well characterized, there remain questions for example with regard to existence of intra-and/or inter-familiar variability (6, 7) and consistency of certain clinical features. Furthermore, the pathogenic mechanism underlying OI type V is still under investigation as well as therapeutic strategies.

Here, we report on the clinical features of 13 Chinese individuals from seven families with molecularly confirmed OI type V in order to investigate inter-and/or intrafamilial variability and consistency of certain clinical features.

## Materials and Methods

### Subjects

**Figure 1:**
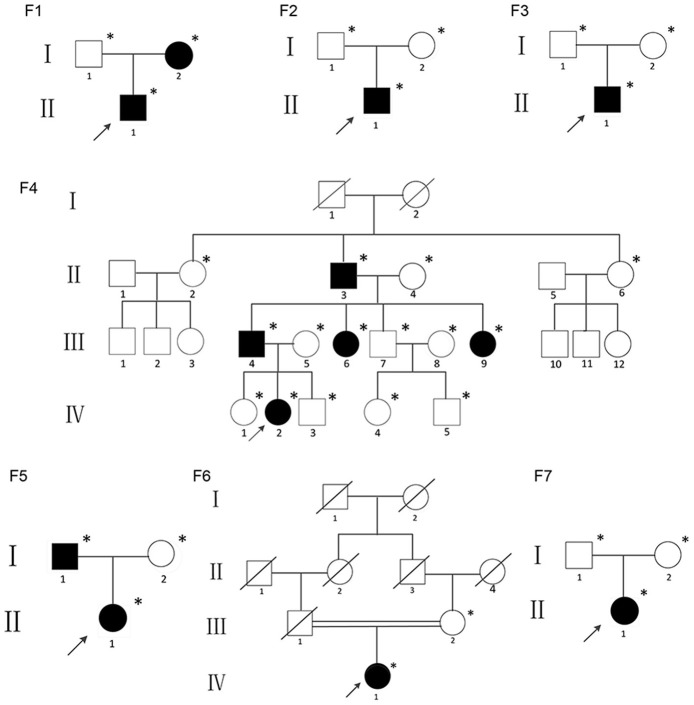
Pedigrees of the individuals described; ^*^DNA available; The black figures represent the individuals who tested positive for the IFITM5 mutation.

The study was approved by the Ethics Committee of the Shanghai Jiao Tong University Affiliated Sixth People's Hospital. Written informed consent was obtained from the families prior to their inclusion in the study. This study included children, adults and adolescents who met the diagnostic criteria for OI type V: (1) history of recurrent fracture; (2) hyperplastic callus formation; (3) radiologically apparent calcification of the forearm; (4) no mutation found on COL1A1 or COL1A2 by Sanger sequencing; and (5) other possible diseases were excluded. None of the individuals had taken bisphosphonates before this study because the patients were recruited from the first-visit outpatient unit of the department of Osteoporosis and Bone Disease of Shanghai Jiao Tong University Affiliated Sixth Peoples Hospital. All the probands and their family members were of Han ethnicity. The pedigrees of the individuals are summarized in Figure 1. Affected individuals form Families 2, 3, 6, and 7 were sporadic cases, and the other nine patients had familial histories. All individuals came from non-consanguineous families except Family six.

### Clinical Evaluation

Physical examination was completed in details. Medical history was collected based on patient's files and information obtained from the patient or their parents. X-ray films of the upper and lower extremities and thoracolumbar vertebrae were also examined. Bone mineral density (BMD) of the lumbar spine (L1-L4), left femoral neck, and total hip was determined in the anterior-posterior direction using Dual-energy X-ray absorptiometry (DXA) (GE Lunar Corp., Madison, WI, USA). The coefficients of variability (CVs) of the lumbar spine, total hip and femoral neck were 1.39, 0.70, and 2.22%, respectively (8). The results were transformed to age-specific Z-scores combining reference data (9–12). Blood samples for biochemistry were collected in a fasting state. Serum total calcium (Ca), phosphate (P), alkaline phosphatase (ALP), intact parathyroid hormone (PTH), 25 hydroxy vitamin D_3_[25(OH)D_3_], β-CrossLaps of type I collagen containing cross-linked C-telopeptide (β-CTX), osteocalcin (OC), erythrocyte sedimentation rate (ESR), and C-reactive protein (C-RP) were measured using standard laboratory methods. Ca, P, ALP, ESR, and C-RP were measured using a HITACHI 7600-020 automatic biochemistry analyzer (Tokyo, Japan). Other biomarkers were measured using the following kits (all from Roche Diagnostics, Mannheim, Switzerland): an intact PTH kit for PTH, a 25-hydroxy vitamin D_3_ kit for 25(OH)D_3_, a β- CrossLaps kit for β-CTX, and an osteocalcin kit for OC. The intra- and inter-assay CVs were reported in previous studies (13–15).

### Mutation Identification and Verification

Next generation sequencing was used for five patients in Family 4 to exclude mutations of other candidate genes for OI because they had an atypical phenotype of OI type V. Sanger sequencing was used to identify those suspected to have OI type V based on their conspicuous hyperplastic callus and to verify all diagnosed individuals.

Genomic DNA was extracted from the peripheral blood of all individuals by standard techniques using a DNA extraction kit (Lifefeng Biotech, Shanghai). Whole-exome sequencing was performed for the five probands of Family four to identify the pathogenic gene. The qualified genomic DNA sample was randomly fragmented by Covaris technology, and the size of the library fragments was mainly distributed between 150 and 250 bp by an AMPure XP-Medium kit (Beckman Coulter, Indiana, USA). Size-selected DNA fragments were amplified by ligation-mediated PCR (LM-PCR) and then purified and hybridized to the exome array for enrichment. The rolling circle amplification (RCA) was performed to produce DNA Nanoballs (DNBs). The captured library was sequenced by the BGISEQ-500 sequencing platform following the manufacturer's instructions. High-throughput sequencing was performed for each captured library to ensure that each sample met the desired average sequencing coverage. Raw image files were processed by BGISEQ-500 base-calling software for base-calling with default parameters, and the sequence data for each individual were generated as paired-end reads (15, 16).

We used Sanger sequencing to assess the DNA samples of probands from Families 1, 2, 3, 5, 6, and 7 with typical symptoms of OI type V and to verify the gene mutation in all available DNA samples from all seven families. Exon 1 and the exon-intron boundary of *IFITM5* were amplified by polymerase chain reaction (PCR). The primer sequences were designed with Primer three software (http://bioinfo.ut.ee/primer3/). The primers used were forward: 5′-AGGGCGACAGGGCTATAAGTGAG-3′ and reverse: 5′-GAAGCCGAGGCAACACAGATTCAGGTAG-3′. Direct sequencing was performed using the BigDye Terminator Cycle Sequencing Ready Reaction Kit, v. 3.1 (Applied Biosystems, Foster, CA), and the sequencing was analyzed with an ABI Prism 3,130 automated sequencer. SNPs were identified using Polyphred (https://droog.gs.washington.edu/polyphred/).

### Systematic Literature Review

Medline and Web of Science were searched, with terms related to OI type V or *IFITM5* from inception to 31 May 2018. Articles on surgical treatment and basic research were excluded from the search.

## Results

### Molecular Diagnosis

**Figure 2:**
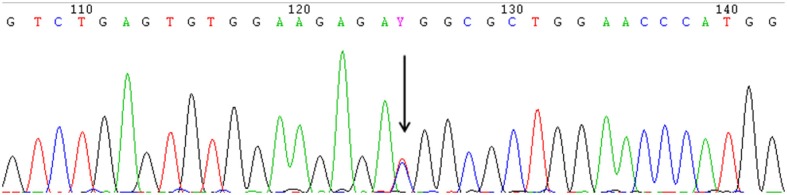
The identical heterozygous c.−14C > T mutation (black arrow) of IFITM5 in all the affected patients with OI type V detected by Sanger sequencing. (This figure is the sequence result for F3II1).

A heterozygous missense mutation in the 5'-UTR of *IFITM5* (c.-14C > T) that was responsible for adding five amino acids at the N-terminus of the bone restricted interferon-inducible transmembrane (IFITM)-like protein (BRIL) was detected in all 13 patients from seven different families (Figure 2).

### Clinical and Radiographic Features

**Figure 3:**
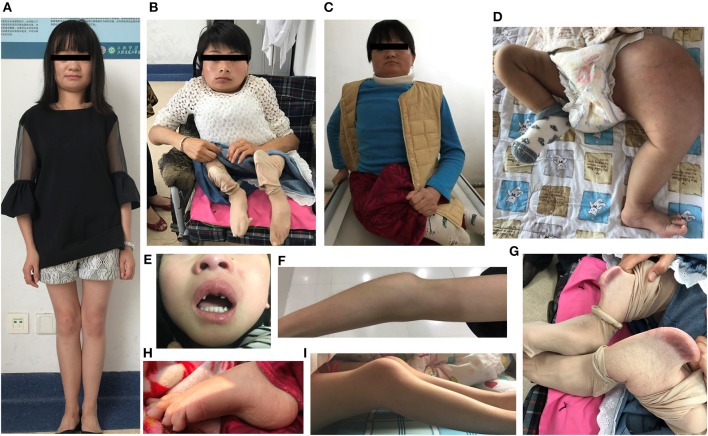
Clinical characteristics of OI type V caused by the IFITM5 c.−14 C> T mutation. **(A–C)** Phenotypic diversity within individuals, and the characteristic facial features: wide set eyes, flat nose, thin lips, broad jaw, and short, wide forehead (F6IV2, F4III6, F4III9). **(D)** Hyperplastic callus (F3II1). **(E)** Dentinogenesis imperfecta (F7II1). **(F)** Large olecranon and coronoid process (F6IV2). **(G)** Saber-like deformity of lower limbs (F4III6). **(H,I)** Joint contracture involving knees and toes (F4IV2, F4III9). Written informed consent for the publication of these images were obtained.

**Figure 4:**
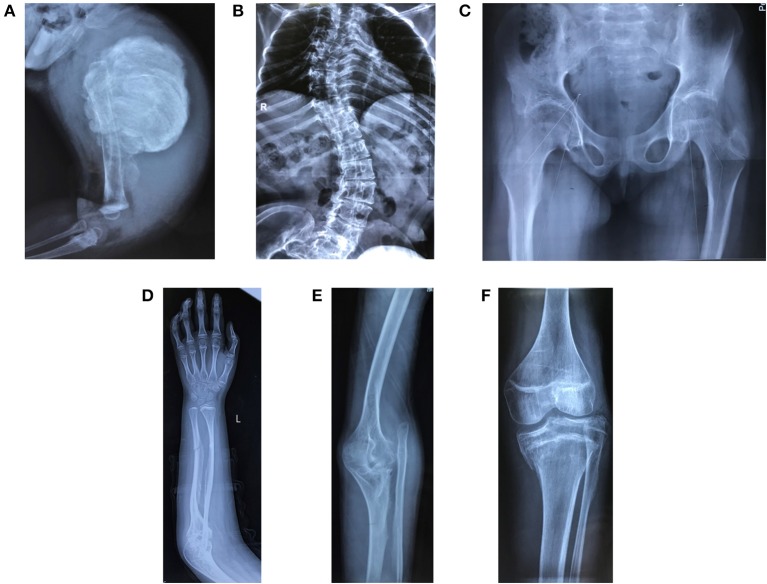
Radiological features of patients in the study. **(A)** Hyperplastic callus (F3II1). **(B)** Severe scoliosis and thin and wavy ribs (F4III9). **(C)** Hip arthritis with effusion and articular surface roughness (F4IV2). **(D)** Ossification of interosseous membrane between the radius and ulna (F2II1). **(E)** Radial head dislocation (F1II1). **(F)** Dense metaphyseal band (F2II1). Written informed consent for the publication of these images was obtained.

The demographic, clinical and radiological findings are summarized in Tables 1, 2. There was considerable clinical variability in the 13 individuals (Figure 3). Not all affected patients suffered frequent fractures. The number of fractures ranged from 0 to 16, and the age of the first fracture ranges from 8 months to 8 years. The Z-score of lumbar spine BMD ranged from −0.5 to −4.6, and the left femoral neck BMD ranged from −0.5 to −5.0. None of the patients presented with blue sclerae or hearing problems, and only one girl (F7II1) was noted to have dentinogenesis imperfecta (Figure 3E). Furthermore, three patients suffered joint contracture, bilateral knees, and toe joints were affected in F4III6 and F4III9, bilateral knees were affected in F4IV2 (Figures 3H–I). The patients recalled that camptodactylia occurred at birth and knee contracture was acquired without any obvious inducement, among which the age of onset was 12 years old, 10 years old, and 8 years in F4III6, F4III9, and F4IV2, respectively. Along with the impact on ambulatory, knee contracture aggravated gradually. Six patients from two families shared suffered unexplained hip arthritis and characteristic facial features including wide set eyes, a flat nose, a broad jaw, a small mouth with thin lips and a short, wide forehead. The onset age of hip arthritis is usually during early childhood and adolescence, and symptoms include squatting exercises difficult and pain in both hips. Moreover, all individuals except the 1-year-old boy in Family 3 had a large olecranon and coronoid process as well as impairment of supination in both forearms, RHD and RUIMO were observed on the radiographs. Additionally, six individuals demonstrated bowing of the long bones (affected femurs [*n* = 3], and tibias and/or fibulas [*n* = 5]), seven individuals suffered severe scoliosis, and nine individuals suffered vertebral compression fractures. A subphyseal-metaphyseal radiodense line was found in four cases by radiological examination (Figure 4).

**Table 1:** Demographic features, fracture incidence and BMD of type V OI patients.

**Patient**	**Sex**	**Age (years)**	**Consanguinity**	**Age of the first fracture (years)**	**Times of fracture**	**Z score of BMD**	
						**L1-L4**	**Femur neck**
F1I2	F	39	_	8	4	−1.9	−0.2
F1II1	M	16	–	4	5	−4.6	N.A
F2II1	M	17	–	3	6	−3.1	−5.0
F3II1	M	1	–	0.7	3	N.A	N.A
F4II3	M	64	–	0	0	−0.5	1.3
F4III4	M	39	–	0	0	−0.9	0.6
F4III6	F	38	–	0.7	14	N.A	N.A
F4III9	F	29	–	0.2	16	N.A	N.A
F4IV2	F	10	–	1.5	6	−0.8	−3.8
F5I1	M	48	–	3	13	N.A	N.A
F5II1	F	14	–	2	6	N.A	−2.8
F6IV2	F	25	+	5	7	−3.1	−0.9
F7II1	F	5	–	0	0	−2.6	N.A

*The Z-scores at lumber spine 1–4, left femoral neck and entire hip of young patients were calculated compared to the age-specific BMD reference values for Chinese children and adolescent*.

**Table 2:** Summary of the clinical and radiological features of 13 individuals from seven families.

	**Suffered individuals**
**CLINICAL FEATURES**	
Blue sclerae	None
Dentinogenesis imperfecta	F7II1
Hearing loss	None
Late fontanel closure (> 2 years)	F4III6, F4III9, F4IV2, F6IV2, F7II1
Characteristic facial features	F4II3, F4III4, F4III6, F4III9, F4IV2, F6IV2
Prominence of the radial styloid process	F1II1, F2II1, F4II3, F4III6, F1I2, F4III4, F4III9, F4IV2, F5I1, F5II1, F6IV2, F7II1
Limited pronation/supination of the forearms	F1II1, F2II1, F4II3, F4III6, F1I2, F4III4, F4III9, F4IV2, F5I1, F5II1, F6IV2, F7II1
Joint contracture	F4III6, F4III9, F4IV2
**RADIOLOGICAL CHARACTERISTIC**	
Bowing of the long bone	F1I2, F1II1, F4II3, F4III6, F4III9, F5I1
Scoliosis	F1I2, F1II1, F4III6, F4III9, F5I1, F5II1, F6IV2
Vertebral compression fracture	F1I2, F1II1, F4III6, F4III9, F5I1, F5II1, F6IV2
Subphyseal-metaphyseal radiodense line	F4IV2, F2II1, F3II1, F7II1
Hyperplastic callus	F1I2, F1II1, F2II1, F3II1, F7II1
Ossification of the interosseous membranes	F1II1, F2II1, F4II3, F4III6, F1I2, F4III4, F4III9, F4IV2, F5I1, F5II1, F6IV2, F7II1
Radial-head dislocation	F1II1, F2II1, F4II3, F4III6, F1I2, F4III4, F4III9, F4IV2, F5I1, F5II1, F6IV2, F7II1
Inflammation of hips	F4II3, F4III4, F4III6, F4III9, F4IV2, F6IV2

**Figure 5:**
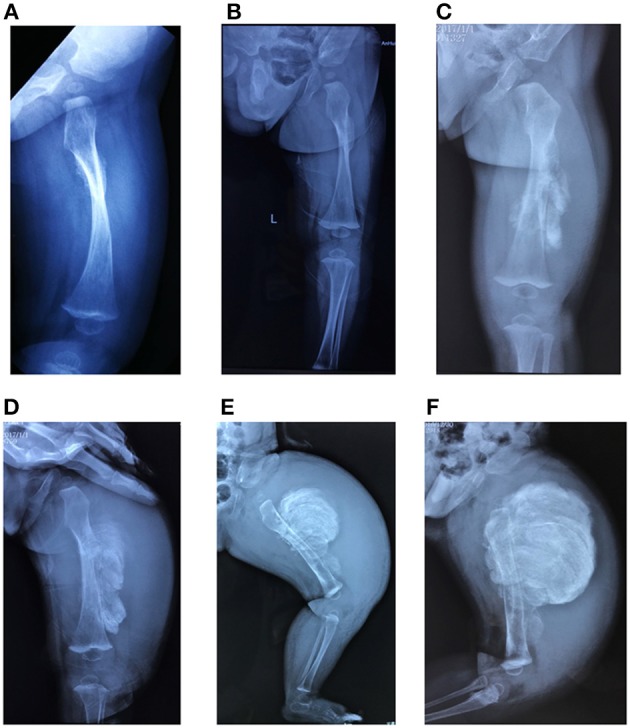
**(A–F)** Lateral X-rays of the left femur captured 15, 30, 45, 60, 75, and 90 days after fractures. Calluses occurred at the fracture site and increased over time (F3II1). Written informed consent for the publication of this image was obtained.

Only five patients experienced typical hyperplastic callus formation, and the calluses formations varied in size and mainly affected the femurs and radius. All the affected individuals had femoral involvement, and only one boy had an additional soy-bean sized callus formation on the right radius. In our cohort, the association between fracture and callus formation was not always clear, as not all callus formations appeared after a fracture (F7II1 had no history of fracture), and the incidence of fractures after which HC developed was irregular (1/4 in F1I1, 1/5 in F1II1, 1/6 in F2II1, and 2/3 in F3II1). Furthermore, in three patients we noticed that HC occurred after complete and oblique fractures and in one patient (F2II1) it occurred after surgical reduction. Generally, a mass arose within a month after a fracture or a surgery and expanded irregularly around the fracture site. We tracked the fractures over 4 months and recorded the duration and size of callus growth but found no pattern. Figure 5 shows the variation in calluses in F3II1.

### Laboratory Findings

Levels of the serum biochemical markers of bone metabolism were generally within the reference range, but serum ESR, and C-RP levels of the probands were higher than normal values during periods of active hyperplastic callus formation (Table 3).

**Table 3:** Laboratory findings in affected members with type V OI.

	**ALP (U/L)**	**Ca (mmol/L)**	**P (mmol/L)**	**PTH (pmol/L)**	**β-CTX (ng/L)**	**OC (ng/ml)**	**25(OH)D (ng/mL)**	**ESR (mm/h)**	**CRP (mg/L)**
Normal value	15–112^a^, 116–380^b^	2.08–2.60^a^, 2.25–2.75^b^	0.80–1.60^a^, 1.29–1.94^b^	15–65	284–576^a^, 500–1860^b^	10–23^a^, 45–125^b^	20–50	0–20	<8
F1I2	47^a^	2.46^a^	1.27^a^	N.A	N.A	N.A	N.A	**97**	N.A
F1II1	275^b^	2.39^b^	1.36^b^	N.A	N.A	N.A	N.A	**108**	N.A
F2II1	**720**^b^	2.43^b^	1.23^b^	1.75	1020.0^b^	43.73^b^	**16.92**	**124**	**10.2**
F3II1	**480**^b^	2.42^b^	1.77^b^	21.44	893.7^b^	31.33^b^	23.79	**114**	**78.7**
F4II3	N.A	N.A	N.A	N.A	N.A	N.A	N.A	N.A	N.A
F4III4	N.A	N.A	N.A	N.A	N.A	N.A	N.A	N.A	N.A
F4III6	54^a^	2.38^a^	1.17^a^	N.A	N.A	N.A	N.A	11	N.A
F4III9	97^a^	2.29^a^	1.00^a^	42.23	356.7^a^	15.96^a^	23.84	6	<3
F4IV2	368^b^	2.68^b^	1.44^b^	18.57	911.2^b^	25.22^b^	29.11	7	<3
F5I1	53^a^	2.34^a^	1.09^a^	31.20	248.3^a^	16.78^a^	18.21	5	N.A
F5II1	184^b^	2.54^b^	1.47^b^	23.23	660.5^b^	58.90^b^	22.73	15	N.A
F6IV2	63^a^	2.36^a^	0.99^a^	58.98	156.1^a^	10.89^a^	20.56	4	<3
F7II1	303^b^	2.61^b^	1.44^b^	15.10	771.6^b^	47.00^b^	38.45	**27**	**21.8**

*Parameters out of the normal range are presented in bold font; NA, not available*.

a *Reference for adults*.

b *Reference for children*.

### Review Analysis

We summarized the phenotype of OI type V in this study and a comparison to the literature (Table 4). The search yielded 56 published articles, of which 14 were retained (2, 3, 5–7, 17–25), a total of 144 cases have been reported. Compared to the literature, The incidence of scoliosis, RUIMO, and RHD in our study were similar to the previous studies. Few extraskeletal symptoms were observed in this study or previous studies. It should be noted, however, dentinogenesis imperfecta, joint contracture, and inflammation of the hips were novel phenotypes that had never been reported before. In contrast, HC was not the most significant trait (38.5 vs. 65.3%) in this study, but the group presented here is a of course smaller.

**Table 4:** Summary of clinical and radiological features with the c.-14C> T mutation in *IFITM5*.

	**This study**	**Percentage of finding in this study**	**References^a^**
Blue sclerae (M/F)	0	0	9.7%
Dentinogenesis imperfecta (M/F)	1	7.7%	0
Hearing loss (M/F)	0	0	1.4%
Bowing of long-bone (M/F)	6 (3/3)	61.5%	N.A
Scoliosis (M/F)	7 (2/5)	53.8%	51.4%
Hyperplastic callus (M/F)	5 (3/2)	38.5%	65.3%
Inflammation of hips (M/F)	6 (2/4)	46.2%	N.A
Joint contracture (M/F)	3 (0/3)	23.1%	N.A
Late fontanel closure (M/F)	5 (0/5)	38.5%	N.A
Characteristic facial features (M/F)	6 (2/4)	46.2%	N.A
Ossification of the interosseous membranes (M/F)	12 (5/7)	92.3%	88.2%
Radial head dislocation (M/F)	12 (5/7)	92.3%	77.1%
Vertebral Compression Fracture (M/F)	9 (3/6)	69.2%	N.A

*NA, not available; M, Male; F, Female*.

a *Reference including 144 individuals from 13 studies analyzed for OI type V phenotype (2, 3, 5–7, 17–25)*.

## Discussion

In 2012 a single pathogenic variant in the *IFITM5* gene (c.-14C> T) appeared to be the genetic cause of OI type V (2, 3). However, the pathogenic mechanism by which the clinical and radiological features of OI type V are caused is currently not clear. In this study, we studied the clinical features of 13 individuals from 7 families with a molecularly confirmed diagnosis of OI type V. In this study, not all patients experienced fractures and low BMD. Specific facial features and joint contractures, as novel clinical features, were reported. We found that radial dislocation and interosseous membrane mineralization were the most representative phenotype, and phenotypic diversity was also common. The presence of hypertrophic callus was infrequent and was rare within families. Furthermore, increased inflammatory markers were observed in subjects with hyperplastic callus. Combined with data from previous 14 studies regarding individuals with OI type V, significant clinical variability, both within and between affected families, is characteristic of OI type V.

Dentinogenesis imperfecta was never reported before in OI type V, but we found that one girl had dentinogenesis imperfecta in our study. Interestingly, she was also the only girl who had HC without any preceding trauma. The puzzling presentation illustrated that trauma was not the only cause of HC. Consistent with 14 previous studies, RHD and RUIMO were found in the majority of OI type V patients, leading to impaired pronation or supination and in the prominence of the radial styloid process, respectively. In addition, compared to the incidences of 1.5, 8.5, and 6.5% for types I, III, and IV, respectively (26), RHD seems to be common in OI type V. The reported prevalence of scoliosis in patients with OI ranged between 39 and 89% (27, 28), and the data were unavailable in Chinese patients until now. Consistent with the mouse model of OI type V (29, 30), bent long bones and scoliosis were found in our subjects. In comparison, scoliosis seems to be more common in females than in males (71.4 in females vs. 2.6% in males). However, no difference in the incidence of long bone deformities was found between females and males. Furthermore, the most susceptible bones seem to be the tibia and fibula.

Similar to Balasubramanian et al. (7), a characteristic facial appearance was observed in six of 13 individuals in this study, but there was an absence of up-turned noses (two patients were reported in their study). Combined with the history of late fontanel closure in our patients, we speculated that they also had craniofacial dysostosis or mineralization disorders. Interestingly, these six patients also had inflammation of the hips. However, 5/6 of the subjects were from the same family, and those clinical features need to be investigated in more patients with OI type V in the future. Joint contractures were found in three patients of a family, which overlapped with the phenotype induced by *FKBP10* and *PLOD2* mutations (31, 32), in patients with these mutations, it usually concerns congenital contractures of the large joints. Reich found decreased collagen expression, suggesting that collagen-related defects were also involved in the pathogenesis of OI type V (5). The overlapped phenotype possibly attributes to the common mechanisms affecting collagen cross-link formation. However, at present, it is still unknown what role *IFITM5* plays in bone formation. The overlap with the clinical spectrum in diverse pathogenic genes of OI portends an unknown underlying link between these genes.

In this study, HC was present in only five patients (38.5%), which is specific but not common for OI type V patients. The difference in percentages are quite large maybe also depending on age, experienced fractures and duration of observation. As early as 1908 (33), HC has been described as a tumor after a fracture; however, 4 years later, the tumor disappeared, which has puzzled the academy for years. In this study, the bone most vulnerable for HC seems to be the femur (100%). The association between fracture or surgery and appearance of callus is irregular, and the occurrence of HC seems to be a random event. Furthermore, it appears that some phenotypic variability in OI type V in our cohort showed sex differences such that females were more prone than males to have skeletal deformities without hyperplastic callus. Previous studies have hypothesized that HC was due to excessive mineralization because a clear positive correlation was observed between *IFITM5* and mineralization *in vitro*, but this relationship was not observed *in vivo* (5, 34). However, we suspected that active inflammation also plays a role in the formation of calluses. In our subjects, we observed that the five affected patients had increased ESR and C-RP (in the available data). This finding has never been reported in other patients with OI type V. Typically, HC presents as a hard, painful, and warm swelling over the affected bone that initially may suggest inflammation. To date, it is still difficult to draw a clear correlation between callus formation and gene mutation. Interestingly, the process was similar to Caffey disease (infantile cortical hyperostosis) (35), which is caused by a single recurrent mutation (c.3040C> T) of the *COL1A1* gene (also responsible for OI type I-IV). Caffey disease is a self-limited acute inflammatory disease characterized by acute inflammation with swelling of soft tissues and hyperostosis of the outer cortical surface in early infancy (36). Laboratory findings in affected patients include elevated ESR and ALP levels along with increased C-RP, suggesting concurrent inflammatory distress (37, 38). Subsequently, Eversole et al. provided a detailed histological analysis of the affected bones with evidence of local inflammation along with subperiosteal new lamellar bone formation (39). These reports are reminiscent of one hallmark in OI type V and implied a relationship with a high rate of collagen turnover (pathological osteogenesis) and inflammation. It is possible that the occurrences of HC and Caffey disease have a similar mechanism. Recently, in an OI type V mouse model, upregulation of Ptgs2 and Nr4a3 also provided some hints toward possible inflammatory pathways (29). On the other hand, previous studies showed that FKBP11 was the only binding partner of IFITM5, and binding IFITM5 to FKBP11 disrupts the binding of CD9 with the FKBP11-CD81-CD9/FPRP complex and leads to immunologically relevant gene expression (40, 41). Therefore, we postulated that *IFITM5* mutation causative of OI type V not only dysregulated the bone formation process but also stimulated the immune response in bone. Strangely, in this study, arthritis of the hip with no clear causative mechanism was found in several family members with OI type V and a sporadic case. We hypothesized that this arthritis may be caused by the same pathogenic mechanism. Although the pathophysiology of Caffey disease is still characterized by a unique clinical conundrum, glucocorticoids, indomethacin, and naproxen, and anti-inflammatory drugs were proven effective at improving inflammation and bone remodelling (42–44). To date, effective treatment for treating HC in OI type V is still missing. Unfortunately, none of the patients underwent bone biopsy in our study, as this is a relatively invasive procedure. We did not detect evidence of local inflammation along with HC. However, our findings highlight that further investigations should be performed to investigate whether anti-inflammatory therapy could be beneficial in patients with OI type V.

## Conclusion

In conclusion, our study describes significant clinical variability and expanded the phenotypic spectrum in Chinese patients diagnosed with OI type V. Also, we present the process of hypertrophic callus formation in detail for the first time, and found evidence of inflammation activity in patients with hyperplastic callus, which provides ideas for research on the pathogenic mechanism of the *IFITM5* mutation causative of OI type V. However, the genotype-phenotype correlation still needs to be investigated in more patients. Moreover, the interactions between genes related to OI are worthy of further study.

## Ethics Statement

This study was carried out in accordance with the recommendations of the ethical standards of the institutional research committee with written informed consent from all subjects. All subjects gave written informed consent in accordance with the Declaration of Helsinki. The protocol was approved by the Ethics Committee of the Shanghai Jiao Tong University Affiliated Sixth People's Hospital.

## Informed Consent

Written informed consent was obtained from each participant before inclusion in the study. For the participants under age 16, written informed consent was obtained from their parents. We also obtained the informed consent for publication of images.

## Author Contributions

Z-LZ and HZ provided the clinical data from the patients, designed the research, and revised the manuscript. ZW helped collect blood samples, and Y-JC summarized the clinical data, analyzed the sequencing data, and drafted the manuscript. All authors read and approved the final manuscript.

### Conflict of Interest Statement

The authors declare that the research was conducted in the absence of any commercial or financial relationships that could be construed as a potential conflict of interest.

## References

[1] Glorieux FH Rauch F Plotkin H Ward L Travers R Roughley P. Type V osteogenesis imperfecta: a new form of brittle bone disease. J Bone Miner Res. (2000) 15:1650–8. 10.1359/jbmr.2000.15.9.1650 10976985

[2] Cho TJ Lee KE Lee SK Song SJ Kim KJ Jeon D. A single recurrent mutation in the 5'-UTR of IFITM5 causes osteogenesis imperfecta type V. Am J Hum Genet. (2012) 91:343–8. 10.1016/j.ajhg.2012.06.005 22863190 PMC3415533

[3] Semler O Garbes L Keupp K Swan D Zimmermann K Becker J. A mutation in the 5'-UTR of IFITM5 creates an in-frame start codon and causes autosomal-dominant osteogenesis imperfecta type V with hyperplastic callus. Am J Hum Genet. (2012) 91:349–57. 10.1016/j.ajhg.2012.06.011 22863195 PMC3415541

[4] Moffatt P Gaumond MH Salois P Sellin K Bessette MC Godin E. Bril: a novel bone-specific modulator of mineralization. J Bone Miner Res. (2008) 23:1497–508. 10.1359/jbmr.080412 18442316

[5] Reich A Bae AS Barnes AM Cabral WA Hinek A Stimec J. Type V OI primary osteoblasts display increased mineralization despite decreased COL1A1 expression. J Clin Endocrinol Metab. (2015) 100:E325–32. 10.1210/jc.2014-3082 25387264 PMC4318905

[6] Shapiro JR Lietman C Grover M Lu JT Nagamani SC Dawson BC. Phenotypic variability of osteogenesis imperfecta type V caused by an IFITM5 mutation. J Bone Miner Res. (2013) 28:1523–30. 10.1002/jbmr.1891 23408678 PMC3688672

[7] Balasubramanian M Parker MJ Dalton A Giunta C Lindert U Peres LC. Genotype-phenotype study in type V osteogenesis imperfecta. Clin Dysmorphol. (2013) 22:93–101. 10.1097/MCD.0b013e32836032f0 23612438

[8] Zhang H He JW Gao G Yue H Yu JB Hu WW Polymorphisms in the HOXD4 gene are not associated with peak bone mineral density in Chinese nuclear families. Acta Pharmacol Sin. (2010) 31:977–83. 10.1038/aps.2010.91 20686522 PMC4007810

[9] Maynard LM Guo SS Chumlea WC Roche AF Wisemandle WA Zeller CM. Total-body and regional bone mineral content and areal bone mineral density in children aged 8–18 y: the Fels Longitudinal Study. Am J Clin Nutr. (1998) 68:1111–7. 10.1093/ajcn/68.5.1111 9808230

[10] Zhang ZQ Ho SC Chen ZQ Zhang CX Chen YM. Reference values of bone mineral density and prevalence of osteoporosis in Chinese adults. Osteoporos Int. (2014) 25:497–507. 10.1007/s00198-013-2418-2 23800746

[11] Xu H Zhao Z Wang H Ding M Zhou A Wang X. Bone mineral density of the spine in 11,898 chinese infants and young children: A Cross-Sectional Study. PLoS ONE. (2013) 8:e82098. 10.1371/journal.pone.0082098 24324752 PMC3855755

[12] Khadilkar AV Sanwalka NJ Chiplonkar SA Khadilkar VV Mughal MZ. Normative data and percentile curves for Dual Energy X-ray Absorptiometry in healthy Indian girls and boys aged 5-17 years. Bone. (2011) 48:810–9. 10.1016/j.bone.2010.12.013 21182992

[13] Lu HK Zhang Z Ke YH He JW Fu WZ Zhang CQ. High prevalence of vitamin D insufficiency in China: relationship with the levels of parathyroid hormone and markers of bone turnover. PLoS ONE. (2012) 7:e47264. 10.1371/journal.pone.0047264 23144810 PMC3493569

[14] He J Zhang H Wang C Zhang Z Yue H Hu W. Associations of serum sclerostin and polymorphisms in the SOST gene with bone mineral density and markers of bone metabolism in postmenopausal Chinese women. J Clin Endocrinol Metab. (2014) 99:E665–73. 10.1210/jc.2013-2086 24423318

[15] Patch AM Nones K Kazakoff SH Newell F Wood S Leonard C. Germline and somatic variant identification using BGISEQ-500 and HiSeq X Ten whole genome sequencing. PLoS ONE. (2018) 13:e0190264. 10.1371/journal.pone.0190264 29320538 PMC5761881

[16] Mak SST Gopalakrishnan S Carøe C Geng C Liu S Sinding MHS. Comparative performance of the BGISEQ-500 vs Illumina HiSeq2500 sequencing platforms for palaeogenomic sequencing. Gigascience. (2017) 6:1. 10.1093/gigascience/gix049 28854615 PMC5570000

[17] Rauch F Moffatt P Cheung M Roughley P Lalic L Lund AM. Osteogenesis imperfecta type V: marked phenotypic variability despite the presence of the IFITM5 c.−14C> T mutation in all patients. J Med Genet. (2013) 50:21–4. 10.1136/jmedgenet-2012-101307 23240094

[18] Liu Y Wang J Ma D Lv F Xu X Xia W. Osteogenesis imperfecta type V: genetic and clinical findings in eleven Chinese patients. Clin Chim Acta. (2016) 462:201–9. 10.1016/j.cca.2016.09.019 27678411

[19] Grover M Campeau PM Lietman CD Lu JT Gibbs RA Schlesinger AE. Osteogenesis imperfecta without features of type V caused by a mutation in the IFITM5 gene. J Bone Miner Res. (2013) 28:2333–7. 10.1002/jbmr.1983 23674381 PMC3800501

[20] Zhang Z Li M He JW Fu WZ Zhang CQ Zhang ZL. Phenotype and genotype analysis of chinese patients with osteogenesis imperfecta type V. PLoS ONE. (2013) 8:e72337. 10.1371/journal.pone.0072337 23977282 PMC3748067

[21] Kim OH Jin DK Kosaki K Kim JW Cho SY Yoo WJ. Osteogenesis imperfecta type V: clinical and radiographic manifestations in mutation confirmed patients. Am J Med Genet A. (2013) 161A:1972–9. 10.1002/ajmg.a.36024 23804581

[22] Takagi M Sato S Hara K Tani C Miyazaki O Nishimura G. A recurrent mutation in the 5'-UTR of IFITM5 causes osteogenesis imperfecta type V. Am J Med Genet A. (2013) 161A:1980–2. 10.1002/ajmg.a.36025 23813632

[23] Guillen-Navarro E Ballesta-Martinez MJ Valencia M Bueno AM Martinez-Glez V Lopez-Gonzalez V. Two mutations in IFITM5 causing distinct forms of osteogenesis imperfecta. Am J Med Genet A. (2014) 164A:1136–42. 10.1002/ajmg.a.36409 24478195

[24] Lazarus S McInerney-Leo AM McKenzie FA Baynam G Broley S Cavan BV. The IFITM5 mutation c.−14C> T results in an elongated transcript expressed in human bone; and causes varying phenotypic severity of osteogenesis imperfecta type V. BMC Musculoskelet Disord. (2014) 15:107. 10.1186/1471-2474-15-107 24674092 PMC3986707

[25] Brizola E Mattos EP Ferrari J Freire PO Germer R Llerena JC Jr. Clinical and Molecular Characterization of Osteogenesis Imperfecta Type V. Mol Syndromol. (2015) 6:164–72. 10.1159/000439506 26648832 PMC4662268

[26] Fassier AM RF Aarabi M Janelle C Fassier F. Radial head dislocation and subluxation in osteogenesis imperfecta. J Bone Joint Surg Am. (2007) 89:2694–704. 10.2106/JBJS.F.01287 18056502

[27] Anissipour AK Hammerberg KW Caudill A Kostiuk T Tarima S Zhao HS. Behavior of scoliosis during growth in children with osteogenesis imperfecta. J Bone Joint Surg Am. (2014) 96:237–43. 10.2106/JBJS.L.01596 24500586 PMC6948836

[28] Sato A Ouellet J Muneta T Glorieux FH Rauch F. Scoliosis in osteogenesis imperfecta caused by COL1A1/COL1A2 mutations—genotype-phenotype correlations and effect of bisphosphonate treatment. Bone. (2016) 86:53–7. 10.1016/j.bone.2016.02.018 26927310

[29] Rauch F Geng Y Lamplugh L Hekmatnejad B Gaumond MH Penney J. Crispr-Cas9 engineered osteogenesis imperfecta type V leads to severe skeletal deformities and perinatal lethality in mice. Bone. (2018) 107:131–42. 10.1016/j.bone.2017.11.013 29174564

[30] Lietman CDMR Munivez E Bertin TK Jiang M-M Chen Y Dawson B. A transgenic mouse model of OI type V supports a neomorphic mechanism of the IFITM5 mutation. J Bone Miner Res. (2015) 30:498–507. 10.1002/jbmr.2363 25251575 PMC4333000

[31] Caparros-Martin JA Aglan MS Temtamy S Otaify GA Valencia M Nevado J Molecular spectrum and differential diagnosis in patients referred with sporadic or autosomal recessive osteogenesis imperfecta. Mol Genet Genomic Med. (2017) 5:28–39. 10.1002/mgg3.257 28116328 PMC5241205

[32] Brenner RE Vetter U Stöss H Müller PK Teller WM. Defective collagen fibril formation and mineralization in osteogenesis imperfecta with congenital joint contractures (Bruck syndrome). Eur J Pediatr. (1993) 152:505–8. 10.1007/BF01955060 8335019

[33] Battle WH Shattock SG. A remarkable case of diffuse cancellous osteoma of the femur, following a fracture, in which similar growths afterwards developed in connection with other bones. Proc R Soc Med. (1908) 1:83. 19973316 10.1177/003591570800101007 PMC2045818

[34] Hanagata N. IFITM5 mutations and osteogenesis imperfecta. J Bone Miner Metab. (2016) 34:123–31. 10.1007/s00774-015-0667-1 26031935

[35] Heyman E Laver J Beer S. Prostaglandin synthetase inhibitor in Caffey disease. J Pediatr. (1982) 101:314. 10.1016/S0022-3476(82)80153-4 6808107

[36] Gensure RC Mäkitie O Barclay C Chan C Depalma SR Bastepe M. A novel COL1A1 mutation in infantile cortical hyperostosis (Caffey disease) expands the spectrum of collagen-related disorders. J Clin Invest. (2005) 115:1250–7. 10.1172/JCI22760 15864348 PMC1087158

[37] Kamoun-Goldrat A Le MM. Infantile cortical hyperostosis (Caffey Disease): a review. J Oral Maxillofac Surg. (2008) 66:2145–50. 10.1016/j.joms.2007.09.007 18848116

[38] Finsterbush A Rang M. Infantile cortical hyperostosis. Follow-up of 29 cases. Acta Orthop Scand. (1975) 46:727. 10.3109/17453677508989258 1106113

[39] Eversole SL Jr Holman GH Robinson RA. Hitherto undescribed characteristics of the pathology of infantile cortical hyperostosis (Caffey's disease). Bull Johns Hopkins Hosp. (1957) 101:80–99. 13446590

[40] Hanagata N Li X. Osteoblast-enriched membrane protein IFITM5 regulates the association of CD9 with an FKBP11-CD81-FPRP complex and stimulates expression of interferoninduced genes. Biochem Biophys Res Commun. (2011) 409:378–84. 10.1016/j.bbrc.2011.04.136 21600883

[41] Tsukamoto T Li X Morita H Minowa T Aizawa T Hanagata N. Role of S-palmitoylation on IFITM5 for the interaction with FKBP11 in osteoblast cells. PLoS ONE. (2013) 8:e75831. 10.1371/journal.pone.0075831 24058703 PMC3776769

[42] Özdemir ÖMA Tancer-Elçi H Polat A Güçtürk I Tepeli E Zeybek S. Familial mutation in Caffey disease with reduced penetrance: a case report. Turk J Pediatr. (2016) 58:650–3. 10.24953/turkjped.2016.06.011 29090879

[43] Thometz JG DiRaimondo CA. A case of recurrent Caffey's disease treated with naproxen. Clin Orthop Relat Res. (1996):304–9. 10.1097/00003086-199602000-00043 8625597

[44] Couper RT McPhee A Morris L. Indomethacin treatment of infantile cortical periostosis in twins. J Paediatr Child Health. (2001) 37:305–8. 10.1046/j.1440-1754.2001.00633.x 11468051

